# Measurement of Personality Structure by the OPD Structure Questionnaire Can Help to Discriminate Between Subtypes of Eating-Disorders

**DOI:** 10.3389/fpsyg.2019.02326

**Published:** 2019-10-17

**Authors:** Jens Rohde, Tobias Hofmann, Barbara Voigt, Matthias Rose, Alexander Obbarius

**Affiliations:** ^1^Department of Psychosomatic Medicine, Center for Internal Medicine and Dermatology, Charité – Universitätsmedizin Berlin, Berlin, Germany; ^2^Quantitative Health Sciences, Outcomes Measurement Science, University of Massachusetts Medical School, Worcester, MA, United States

**Keywords:** eating disorder, anorexia nervosa, purging type, restricting type, bulimia nervosa, operationalized psychodynamic diagnosis, personality structure

## Abstract

**Background:**

Differentiation between purging type (AN-P) and restricting type (AN-R) is common in anorexia nervosa (AN) and relevant for clinical practice. However, differences of personality pathology in eating disorders (ED) and their subtypes, which can be captured by the operationalized psychodynamic diagnosis (OPD) system, have not been systematically investigated to date.

**Objectives:**

The aim of this study was to explore differences in personality structure between the subtypes of AN and bulimia nervosa (BN) using the OPD structure questionnaire (OPD-SQ). In addition, the ability of the instrument to support the classification of eating disorders was examined.

**Materials and Methods:**

We conducted a retrospective, exploratory study in a subset sample of a larger validation study. The OPD-SQ had been collected from *n* = 60 patients with AN or BN. Patients were assigned to the ED groups by clinical assessment. Statistical analyses included multivariate analysis of variance (MANOVA) and discriminant analysis.

**Results:**

Differences between ED groups were observed on 5 OPD-SQ main scales and 9 subscales, as well as on the global scale. AN-P patients demonstrated the lowest personality structure on most of the main scales and subscales, whereas AN-R patients showed a higher personality structure level as compared to both BN and AN-P patients. The OPD-SQ scales with the largest differences include self-perception, object perception, and attachment to internal objects. Discriminant analysis resulted in satisfactory assignment to ED groups by OPD-SQ subscales.

**Conclusions:**

Personality structure was found to be less developed in patients with BN and AN-P as compared to patients with AN-R. Although the results have to be proven in larger prospective studies, these results suggest that the OPD-SQ may be used to support the clinical assessment and classification in patients with EDs.

## Introduction

Treatment of eating disorders (ED) such as anorexia nervosa (AN) and bulimia nervosa (BN) is challenging (Vall and Wade, 2015). Lifetime-prevalence of AN and BN in women is 0.9 and 1.5%, respectively (Treasure, 2016), and about one-third of AN patients are seriously affected and stay chronically ill (Treasure, 2016). In addition, as compared to other mental disorders, mortality rate of AN is high (Kask et al., 2016). “Sub-threshold” disturbance in personality functioning or manifest personality disorders (PD) are frequent comorbidities (Martinussen et al., 2017), which contribute to the difficulties during treatment and may support the persistence of the ED in some patients (Farstad et al., 2016). For example, the evidence-based cognitive-interpersonal maintenance model of anorexia nervosa refers to obsessive compulsive features and anxious avoidance as contributing factors (Schmidt and Treasure, 2006). However, there is still a lack of empirical research about the extent of personality functioning across different types of EDs and especially the comparison of those between different EDs.

A range of studies in the empirical literature reported relationships between PD diagnoses and different types of EDs (see Rosenvinge et al., 2000 or Farstad et al., 2016 for an overview). Restricting type AN (AN-R) was most commonly associated with obsessive-compulsive, avoidant, and dependent PD. Obsessive-compulsive, avoidant, borderline, dependent, and paranoid PDs were found to be most prevalent in both purging type AN (AN-P) and BN. Patients with Binge-eating disorders (BED) showed a high prevalence of avoidant, obsessive-compulsive, and paranoid PDs (Farstad et al., 2016). Thus, avoidant and obsessive-compulsive PDs were most frequently diagnosed among AN-R and BED and the authors concluded that these patients might tend to perceive more concern with acceptance and approval, fear of criticism and rejection, as well as perfectionism. As borderline and paranoid PD were most commonly found as a comorbidity in BN and AN-P, these patients might have greater levels of emotion dysregulation, impulsivity, and suspiciousness of others (Farstad et al., 2016). A few studies described personality pathology across groups of EDs based on cluster analyses (Brown et al., 2011; Waller et al., 2013; Gabriel and Waller, 2014; Peterhansel et al., 2017). For example, one study in 214 eating-disordered women observed three clusters including a group with low levels of personality disturbance overall, a second group with high levels of cognitions with anxiety-based personality disturbance, and a third group with high levels of all personality disturbance dimensions (Waller et al., 2013). However, further research is needed for in-depth investigation of differences of personality functioning across EDs.

To date, existing categorical classifications of personality disorder impeded the characterization of different patterns of affected personality aspects of patients (World Health Organization [WHO], 1993; American Psychiatric Association, 2013). In recent years, a growing number of researchers and clinicians favor a dimensional or a composite categorical-dimensional approach for personality pathology rather than a categorical one (Al-Dajani et al., 2016), which is supported by empirical findings. For example, there is evidence that distinct PD categories such as borderline, histrionic, or dependent are not fully represented by exploratory research (Haslam et al., 2012). In addition, conventional classifications do not include common criteria for personality pathology leading to a lack of conceptual clarity about common aspects of personality disorders as compared to a “healthy” personality or other mental disorders (Tyrer et al., 2011). Therefore, a mixed categorical-dimensional classification was suggested for the latest revisions of the Diagnostic and Statistical Manual of Mental Disorders (DSM) and the International Classification of Diseases (ICD). This alternative approach was included in the DSM-5 appendix for further investigation (American Psychiatric Association, 2013). Furthermore, in the recently released ICD-11 classification, three different PD levels have been implemented as primary method to determine the extent of personality functioning rather than PD categories (World Health Organization [WHO], 2018). In summary, these new approaches allow a more detailed characterization and comparison of patients.

However, due to the recent change of PD classifications, there is a growing number of tools available that allow a dimensional assessment of personality functioning (Wright and Simms, 2014; Bender et al., 2018). A system, that is well-established in German-speaking countries is a composite system of a unidimensional scale based on eight dimensions and 24 subdimensions and was proposed by the operationalized psychodynamic diagnosis (OPD) research group (OPD Task Force, 2008). The “personality structure” construct includes a broad range of personality facets and is very similar to the levels of personality functioning (LPFS) as introduced in DSM-5 (Zimmermann et al., 2012). The 95-item OPD structure questionnaire (OPD-SQ) was derived to allow economic patient-centered self-report assessment (Ehrenthal et al., 2012). The OPD-SQ showed a correlation of *r* = 0.62 with the expert rating (Dinger et al., 2014) and a growing number of studies confirmed the validity and reliability of the instrument in various clinical samples (Ehrenthal et al., 2012; Zimmermann et al., 2015; König et al., 2016). In addition, the instrument has been shown to correlate with similar instruments assessing personality functioning (König et al., 2016). Over many decades, patients with affected personality have been characterized with words such as “borderline,” “narcissistic,” “psychopathic” etc., supported by classification systems (Sheehan et al., 2016). However, the neutral language of the OPD is in-line with recent developments in refining the PD classifications and can therefore help protect patients from stigmatization (OPD Task Force, 2008). Thus, although international validation studies are desirable, this instrument seems to be suitable for the evaluation of personality functioning in patients with EDs.

The aim of the present study was to explore differences in personality functioning between AN-R, AN-P and BN. The OPD-SQ was used to allow a detailed description of the patients’ personality facets. In addition, the ability of the instrument to support the classification of eating disorders was examined.

Although the terminology which is used to describe affected personality is heterogeneous across the literature, we use “personality functioning” throughout the text to indicate personality difficulties which do not necessarily exceed the threshold for classical categorical “personality disorders.” In contrast, “personality structure” is used to describe the OPD structure dimension (see section “Operationalized Psychodynamic Diagnosis-Structure Questionnaire”). “Personality pathology” is used to describe disordered personality in general.

## Materials and Methods

### Sample and Setting

A retrospective analysis was carried out in patients with eating disorders. Patients from the Department of Psychosomatic Medicine, Charité – Universitätsmedizin Berlin with clinical diagnoses of AN or BN according to ICD-10 were included during the first week of inpatient psychosomatic treatment or at outpatient visits (American Psychiatric Association, 2013). Psychometric information was gathered electronically with smartphones along with the clinical routine assessment between 2012 and 2017. Data from *N* = 1235 patients was gathered during this period of time, see Obbarius et al. (2018) for a detailed description of patient characteristics. A subset of *N* = 60 patients were found to have AN (typical or atypical) or BN and were included in this study. Patients were assigned to BN, AN-R, and AN-P groups using ICD-10 criteria. Patients with atypical AN were included, if one diagnostic criterion (such as BMI ≤ 17.5 kg/m^2^) was not fulfilled. Clinical information was obtained from doctor’s letters and was supported by results from the Eating disorder inventory 2, EDI-2.

### Instruments

#### Operationalized Psychodynamic Diagnosis-Structure Questionnaire (OPD-SQ)

The OPD-SQ is an instrument for assessment of personality functioning according to the Levels of Structural Integration Axis (LSIA) of the operationalized psychodynamic diagnosis (OPD) system (OPD Task Force, 2008). The multiaxial OPD system was developed as a semi-structured interview and is broadly used by therapists in German-speaking countries as an add-on to classification systems such as ICD or DSM. The OPD system relies on psychodynamic theory and allows a very fine-grained assessment of patients’ psychopathology including the patient’s experience of illness (axis I) interpersonal relations (axis II), intrapsychic conflicts (axis III) and personality structure (axis IV). These four axes are accompanied by the descriptive classification of mental and psychosomatic disorders (axis V) according to the ICD or DSM.

A few years ago, the OPD-SQ was developed as a self-report measure to allow for bedside assessment of personality structure (OPD axis IV). It consists of 95 items to be rated on a 5-point Likert scale, including the eight main scales and 21 of the 24 subscales of the OPD (see Table 1 for an overview). All scales are describing aspects of mental functions for the regulation of the self and its relationships to internal and external objects (OPD Task Force, 2008). The main scales consist of the four dimensions perception, regulation, communication and attachment. Every dimension is represented by two main scales which refer to the self and others (e.g., self-perception and object-perception). Each main scale is assembled of 2–3 subscales which yield individual scores for the level of structural integration from 0 (highly integrated = high personality functioning) to 4 (disintegrated = low personality functioning) leading to a complex profile of personality structure. The mean score of all subscales serves as a global severity index. Higher scores indicate greater impairment and lower scores indicate less impairment. Validation studies show satisfying psychometric properties including reliability (Cronbach’s α = 0.96) and construct validity (Ehrenthal et al., 2012; König et al., 2016). For example, the subscales of the OPD-SQ show correlations in the expected directions with other measures of personality and attachment (König et al., 2016), number of DSM-IV PD diagnoses (Ehrenthal et al., 2012), and expert-ratings of the LSIA (Dinger et al., 2014; König et al., 2016).

**TABLE 1:** Main and subscales of the OPD structure questionnaire (OPD-SF).

	**Self**	**Object**
Perception/cognition	**Self-perception** - Self-reflection - Affect differentiation - Identity	**Object-perception** - Self-object differentiation - Whole object perception - Realistic object perception
Regulation	**Self-regulation** - Affect tolerance - Impulse control - Regulation of self-esteem	**Regulation of relationships** - Balancing interests in relationships - Anticipation
Communication	**Internal communication** - Experiencing affect - Use of fantasies - Bodily self	**External communication** - Making contact - Communicating affect - Empathy
Attachment	**Attachment to internal objects** - Internalization - Use of introjects	**Attachment to external objects** - Accepting help - Detaching from relationships

#### Eating Disorder Inventory (EDI-2)

The second version of the Eating Disorder Inventory, the EDI-2 (Paul and Thiel, 2005), is a self-report instrument for assessing eating behavior and associated psychological characteristics among patients with AN and BN. It includes 91 items resulting in 11 scales covering a broad range of eating pathology such as “bulimia,” “body dissatisfaction” and “drive for thinness.” All scales showed significant test–retest correlations between 0.81 and 0.89 (Thiel and Paul, 2006), and most of them had acceptable specificity and sensitivity for the detection of abnormal eating behavior (Paul and Thiel, 2005). In addition, the EDI-2 was successfully used to support the discrimination between AN-R and AN-P in critical cases (Thiel et al., 1997).

### Statistical Analyses

All descriptive und inferential analyses were carried out with IBM SPSS^TM^ Statistics Version 24 (IBM Corp., Armonk, NY, United States). A series of one-way ANOVAs were conducted to compare the ED groups (AN-R, AN-P, BN) in terms of sociodemographic variables (age, gender, education level, and marital status), setting (outpatient vs. inpatient) and BMI if the variables complied with the assumptions of the analysis of variance (Levene’s and Shapiro-Wilk tests). Otherwise, non-parametric Mann-Whitney *U* tests were performed. An ANOVA was calculated to evaluate differences of the OPD-SQ global scale across ED groups. In order to compare the OPD-SQ scales and subscales across ED groups multivariate analyses of variances (MANOVA) were used rather than multiple ANOVAs to allow for effects between scales and subscales within the models. Four most common test statistic criterions are provided for MANOVAs: Pillai’s Trace V, Wilks’ Λ, Hotelling’s T2, Roy’s largest root (Olson, 1976). *Post hoc* tests with Bonferroni correction were performed to control for type one error. η^2^ was calculated by dividing between-group sum of squares by total sum of squares and is thus identical with the *R*^2^ from multiple linear regressions. A η^2^ ≥ 0.01 is regarded as small effect and a η^2^ ≥ 0.14 is regarded as large effect (Gill, 2000). All effect sizes were converted from η^2^ to Cohen’s *d* for better comparability (Borenstein et al., 2011).

Furthermore, a discriminant analysis was performed to quantify the discriminative value of each subscale of the OPD-SQ in their ability to assign the patients to the three ED groups. The analysis is used to investigate how a set of groups can be best separated using several predictors (McLachlan, 2004). It creates linear combinations of predictors, taking a new latent variable as a basis for discriminant functions. These new created functions maximize the difference between the given set of groups. Wilks’ Λ is a measure of how well each function separates cases into groups with values between 0 and 1. Smaller values of Wilks’ Λ indicate greater discriminatory ability of a function.

## Results

### Sample

*N* = 60 patients with complete datasets were included for analyses. Two-third were outpatients (*n* = 42) and one-third underwent inpatient treatment (*n* = 18). Sample characteristics are shown in Table 2. More than half had an AN-R diagnosis and 25% a BN diagnosis. The mean age was 27.6 years, almost all participants were female (*n* = 57) and about 16% of the participants were married or lived in a permanent relationship. There was no significant difference between in- and outpatients on the OPD global scale (*p* = 0.527).

**TABLE 2:** Sample characteristics.

	**AN-R**	**AN-P**	**BN**	
	***n* = 36**	***n* = 9**	***n* = 15**	***p***
Age in years M (SD)	27 (8.90)	28 (6.97)	30 (10.68)	0.55
range in years	18–58	20–41	18–56	
Gender female N (%)	33 (92)	9 (100)	14 (93)	0.68
Marital status N (%)				0.14
single	32 (89)	8 (89)	10 (67)	
with partner	4 (11)	1 (11)	5 (33)	
Educational level N (%)				0.61
university entrance diploma	17 (47)	4 (44)	3 (20)	
certificate of secondary education	9 (25)	2 (22)	7 (47)	
certificate of primary or lower secondary education	5 (14)	2 (22)	3 (20)	
without	5 (14)	1 (11)	2 (13)	
BMI in kg/m^2^ M (SD)	15.4 (2.04)	15.6 (2.61)	20.7 (3.21)	< 0.01
range of BMI	12.3–20.1	11.2–19.0	17.6–29.5	
Setting N (%)				0.17
outpatient	26 (72)	4 (44)	12 (80)	
inpatient	10 (28)	5 (56)	3 (20)	

*AN-R = restricting type anorexia nervosa, AN-P = purging type anorexia nervosa, BMI = body mass index, BN = bulimia nervosa M = mean, *N* = number, *p* = *p*-value, statistical significance; SD = standard deviation.*

### Differences Between Types of EDs

The comparison of the demographic variables showed no significant differences between ED groups (AN-R, AN-P, BN) apart from BMI (*p* < 0.05). As one would expect, BMI was significantly lower in AN-R and AN-P than in BN. However, we did decide to not include BMI as covariate in our primary analysis as it is not directly associated with the dependent variable (OPD scales and subscales) but rather with the independent variable (ED group) and could thus reduce group differences in the analysis (Miller and Chapman, 2001). Testing the ANOVA assumptions indicated that four scales (“Whole object perception,” “Object perception,” “Regulation of relationships” and “Experiencing affect”) showed a lack of homoscedasticity or normal distribution and non-parametric comparison of groups was applied.

**FIGURE 1:**
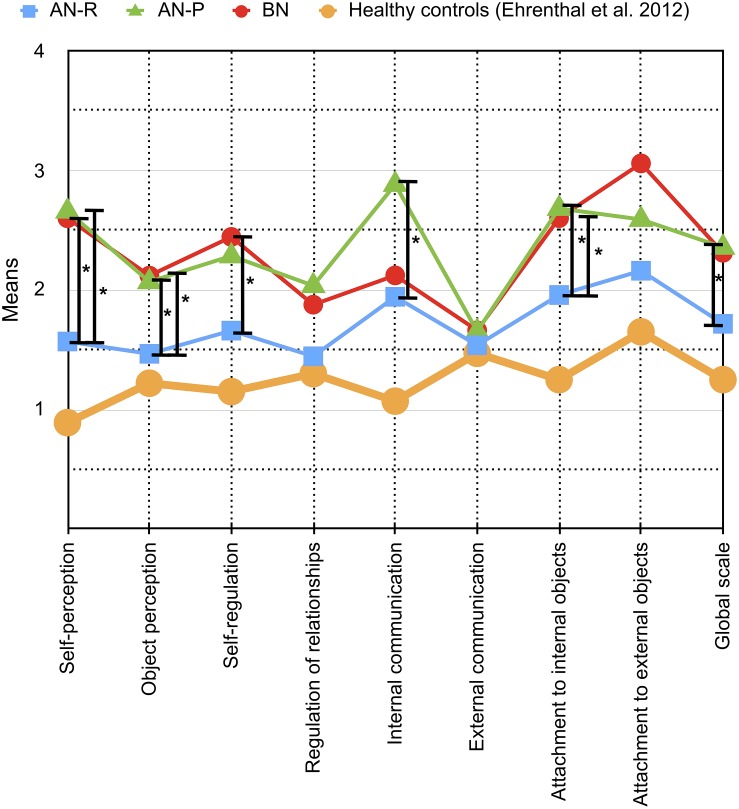
Means and differences of OPD-SQ main scales (*x*-axis) across different eating disorder (ED) groups are depicted in the figure: Purging-type anorexia nervosa (AN-P, green line) vs. restricting-type anorexia nervosa (AN-R, blue line) vs. bulimia nervosa (BN, red line). The extent of personality structure (*y*-axis) varies between 0 (= no disturbance) and 4 (= severe disturbance). Significant differences between ED groups are tagged with an asterisk (^∗^). To allow for comparison with a healthy sample, data from the validation study by Ehrenthal et al. (2012) of patients without psychological treatment are shown in addition (yellow line).

**FIGURE 2:**
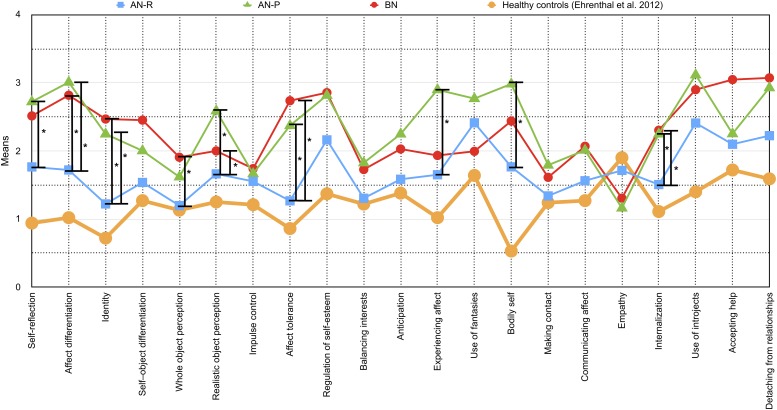
Means and differences of OPD-SQ subscales (*x*-axis) across different eating disorder (ED) groups are depicted in the figure: Purging-type anorexia nervosa (AN-P, green line) vs. restricting-type anorexia nervosa (AN-R, blue line) vs. bulimia nervosa (BN, red line). The extent of personality structure (*y*-axis) varies between 0 (= no disturbance) and 4 (= severe disturbance). Significant differences between ED groups are tagged with an asterisk (^∗^). To allow for comparison with a healthy sample, data from the validation study by Ehrenthal et al. (2012) of patients without psychological treatment are shown in addition (yellow line).

The ANOVA of the OPD-SQ global scale across ED groups indicated significant differences [*F*(2,57) = 6.26, *p* < 0.01, Figure 1]. The MANOVA model of the OPD-SQ scales revealed significant differences between the three ED groups (Table 3). Only two of four statistical criteria indicated significant differences between ED groups in the OPD-SQ subscale MANOVA model (Table 3). Nine subscales, five main scales and the global scale differed in *post hoc* pairwise comparisons (Figures 1, 2). For more comprehensive presentation, results of *post hoc* test differences were divided into three groups:

**TABLE 3:** Main effects in the MANOVA models for the comparison of OPD-SQ scales and subscales across ED groups.

**Model**	**Criterion**	**Statistic**	***F***	***p***	**η^2^**	**Cohen’s *d***
OPD-SQ scales	Pillai’s Trace V	0.570	2.543	0.002	0.285	1.263
	Wilks’ Λ	0.490	2.679	0.001	0.300	1.309
	Hotelling’s T^2^	0.918	2.811	0.001	0.315	1.356
	Roy’s largest root	0.755	4.810	0.000	0.430	1.737
OPD-SQ subscales	Pillai’s Trace V	0.878	1.416	0.094	0.439	1.769
	Wilks’ Λ	0.291	1.506	0.062	0.461	1.850
	Hotelling’s T^2^	1.860	1.594	0.041	0.482	1.929
	Roy’s largest root	1.464	2.649	0.004	0.594	2.419

*ED = eating disorder, η^2^ = Partial eta square, *F* = Multivariate *F*-statistics, MANOVA = Multivariate analysis of variance, OPD-SQ = OPD structure questionnaire, *p* = significance.*

#### Differences Between AN-R and Both AN-P and BN

In the first group of scales AN-R differed significantly from both AN-P and BN. It included the subscales “affect differentiation” (*d* = 1.081, “identity” (*d* = 1.000), “realistic object perception” (*d* = 1.201), “affect tolerance” (*d* = 1.288), “internalization” (*d* = 0.714) as well as the main scales “self-perception” (*d* = 1.078), “object perception” (*d* = 0.139) and “attachment to internal objects” (*d* = 0.969). Patients diagnosed with AN-R scored significantly lower on these scales than patients of the other groups, whose means indicated a moderate to severe impairment.

#### Differences Between AN-R and BN

The scales in the second group varied significantly between AN-R and BN. It consisted of the subscale “whole object perception” (*d* = 0.773) and the main scale “self-regulation” (*d* = 0.956). BN patients showed a greater impairment than patients with AN-R. The AN-P group scored between them and did not differ significantly.

#### Differences Between AN-R and AN-P

In the third group of scales, the subtypes of AN were found to be different from each other, including the subscales “self-reflection” (*d* = 0.787), “experiencing affect” (*d* = 0.834) and “bodily self” (*d* = 0.773), as well as the main scale “internal communication” (*d* = 0.756). It also includes the “global scale” of the OPD-SQ (*p* < 0.001, *d* = 0.937). Patients with AN-R again scored lower than AN-P. Although the conventional level of significance was missed for the difference of AN-R and BN on the global scale *p* = 0.055, BN patients demonstrated a similar mean level as AN-P patients.

### Discrimination Between Types of EDs

Based on the results of the MANOVA the nine subscales that exhibited significant differences between the ED subgroups were included in the discriminant analysis. Since the main scales are derived from the subscales, they were not added to the model.

The analysis revealed two discriminant functions. The first function explained 82.5% of the variance (canonical *R*^2^ = 0.478), whereas the second function explained only 17.5% (canonical *R*^2^ = 0.163). In combination, these discriminant functions significantly differentiated between ED groups (Λ = 0.435, χ^2^ = 43.649, *p* = 0.002). However, when the first function was removed, the second function alone did not significantly discriminate between ED groups (Λ = 0.836, χ^2^ = 9,378, *p* = 0.0403).

**FIGURE 3:**
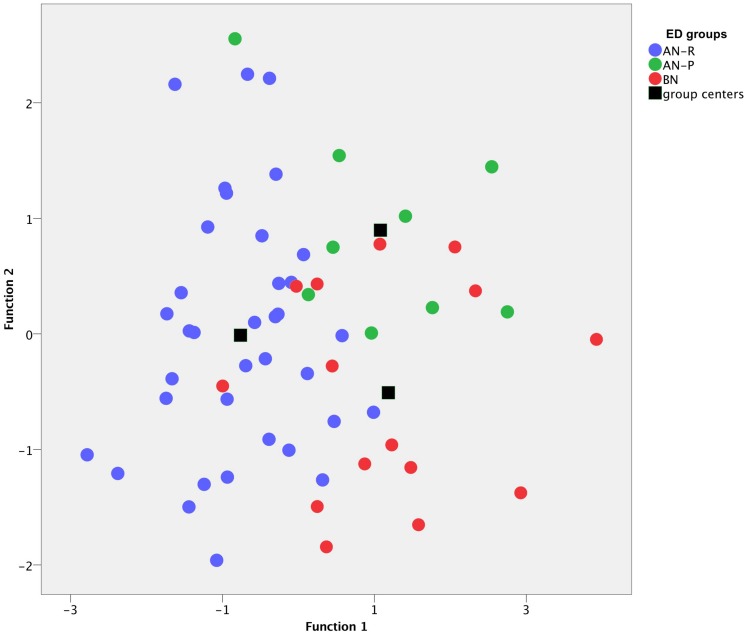
Discriminant analysis of three eating disorder (ED) groups on the basis of two functions (*x*-axis and *y*-axis) including 10 OPD structure questionnaire (OPD-SQ) subscales. Each dot represents a patient, ED diagnoses are color-coded: Purging-type anorexia nervosa (AN-P, green dots) vs. restricting-type anorexia nervosa (AN-R, blue dots) vs. bulimia nervosa (BN, red dots). Black boxes highlight group centers (means of each group), which are separated from each other.

For better visualization, the two functions were included as *x*- and *y*-axis in a 2-dimensional coordinate system (“discriminate function plot” Figure 3). It illustrates that the first function, which is shown on the *x*-axis, discriminated the AN-R group from the other groups (BN and AN-P). The second function, which is shown on the *y*-axis, discriminates the AN-P group from the other groups (BN and AN-R). Patients with AN-R are mostly displayed on the left side, whereas patients with AN-P are displayed on the upper half (Figure 3). The correlations between the analyzed scales and the discriminant functions (Table 4) revealed that “affect tolerance” (*r* = 0.670), “realistic object perception” (*r* = 0.606) and “affect differentiation” (*r* = 0.542) showed close relation with the first function, “experiencing affect” (*r* = 0.637) and “bodily self” (*r* = 0.582) indicated close relation with the second function (Table 4).

**TABLE 4:** Correlation of two discriminant functions with nine OPD-SQ main scales.

	**Correlations with functions (*r*)**	
**Function**	**1**	**2**
Affect tolerance	0.670	–0.056
Realistic object perception	0.606	0.341
Affect differentiation	0.542	0.334
Identity	0.515	0.168
Internalization	0.486	0.111
Self-perception	0.324	–0.003
Experiencing affect	0.320	0.637
Bodily self	0.301	0.582
Self-reflection	0.369	0.385

*r = correlation coefficient.*

## Discussion

In this exploratory, retrospective study, we observed a range of variations of OPD personality structure facets between AN-R, AN-P, and BN. Although the size of the total sample of *N* = 60 was rather low, we were able to find significant differences on nine subscales and five main scales which were supported by high effect sizes. Furthermore, the combination of nine OPD-SQ subscales proved to be suitable for the discrimination of these ED subtypes. Taken together, the personality structure level (OPD-SQ global scale) was higher (better personality functioning) in AN-R patients as compared to AN-P patients. However, although the mean level of BN patients was almost as high as the level of AN-P patients, the conventional level of significance was missed. Similar differences were found for scales reflecting perception of the self and the object, as well as attachment to internal and external objects. Very large effects were found on the self-perception subscales including self-reflection, affect differentiation, and identity (Figures 1, 2). Consequently, health-care professionals such as psychotherapists, social workers, and physicians should be aware of interpersonal consequences which may result from those difficulties and which may complicate routine care as well as individual treatments in patients with AN-P and BN (Pham-Scottez et al., 2012).

With regard to the treatment of impaired personality functioning, these exploratory findings might support the assumption that patients with AN-P should not receive the same treatments as AN-R patients, but rather similar treatments as BN patients. For example, some BN patients, especially with a comorbid PD, can successfully be treated with dialectical behavioral therapy (Kroger et al., 2010) or transference-focused psychotherapy (Levy et al., 2006). In addition, in the light of the differences in personality structure levels, the focus of psychotherapy in eating disorders may be suited preferably to the personality functioning of different ED groups. For example, AN-P patients may benefit – as compared to AN-R patients – from psychotherapeutic interventions pointing at affect differentiation and self-reflection. However, due to the exploratory character of the study, these statements should not be regarded as a recommendation for clinical practice as they have to be confirmed in further research.

BMI was not included as a covariate in the MANOVA models as it was not directly connected with the dependent variable (OPD scales and subscales). However, one might assume an indirect effect of BMI through affected brain structure and alteration of brain functions caused by low weight. For example, changes in the reward system (Scharner and Stengel, 2019) or cognitive rigidity (Abbate-Daga et al., 2011) have been described in the literature to be related to severe anorexia. Therefore, we think that the inclusion of BMI as a covariate might have been justifiable although between-group differences may have been reduced. To evaluate possible great differences multivariate analysis of covariance (MANCOVA) models including BMI were calculated, too. However, these analyses did only result in a few minor differences. Four (sub)scales did not reach statistical significance in terms of group differences as compared to prior analyses, while two other (sub)scales did now reach statistical significance (data not shown). Taken together, general differences between OPD-SQ levels of ED groups did not change and statistically strong differences such as on self-perception and object perception scales and subscales did not change, either. Thus, we concluded that both series of analyses (with and without BMI as covariate) yielded very similar results.

Our current study investigated differences of personality structure facets across anorexia nervosa subtypes and bulimia nervosa. We are not aware of previous empirical investigations on the relationship of eating disorders and the level of personality functioning. Available research did either describe personality differences based on common personality models such as the Big Five Personality Model (Claes et al., 2006) or did deal with comorbid personality disorders (PD) as categorical entities rather than dimensional aspects (Westen and Harnden-Fischer, 2001; Thompson-Brenner et al., 2008; Krug et al., 2011). A range of studies found that AN-P and BN are associated with greater levels of emotion dysregulation, impulsivity, and suspiciousness of others (Sansone et al., 2004; Cassin and von Ranson, 2005; Farstad et al., 2016). Our findings are partly in line with these findings as AN-P and BN patients displayed lower scores on the “self-regulation” and “regulation of relationships” subscales. However, statistical significance was not reached. Impulsivity is reflected by the OPD-SQ subscales “impulse control” and “affect tolerance,” and although “impulse control” did not show differences between groups, “affect tolerance” was shown to be significantly better in AN-R patients as compared to BN and AN-P patients. Although suspiciousness of others is a key feature of PDs (Kellett and Hardy, 2014), it does not have an equivalent item or scale in the OPD-SQ instrument. However, “external communication” has the largest overlap with this feature. Apart from the psychodynamic theoretical background of the OPD-SQ, this fact is due to the intention of the developers of the OPD system to describe abilities of personality skills rather than impairments (Cierpka et al., 2007). Previous research reported that prevalence rates of all PD clusters were lower among AN-R patients as compared to BN and AN-P patients (Sansone et al., 2004; Cassin and von Ranson, 2005; Farstad et al., 2016). Our results are also in line with these findings as the overall level of personality structure was lower in AN-R patients as compared to both, BN and AN-P patients.

In addition, these findings also support a range of clinical observations and case reports, which describe AN-P as most severely ill and most resistant to therapy when compared with both AN-R and BN patients (Fichter, 1985; Murakami et al., 2002; Santonastaso et al., 2006). Our results are also coherent with observations from psychodynamically oriented therapists in terms of self-perception and self-regulation of patients with eating disorders: For example, patients who actively show counter-regulating or weight-reducing behavior (BN and AN-P) showed impairments in affect differentiation and identity as compared to the AN-R group (Ettl, 2013). In addition, a lower ability for affect tolerance in BN as compared to AN-R has also been reported before (Jeammet, 1997; Murakami et al., 2002; Stasch et al., 2014).

### Discrimination of Patients With BN and Subtypes of AN

The discriminant analyses demonstrated that both the subscales and the main scales are able to discriminate well between the three ED groups. However, the subscales performed better, which is most likely due to the loss of information during the formation of the main scales. More precisely, the combination of nine subscales facilitates the distinction of all three ED groups from each other satisfactorily, which may allow for clinical application in the future (see below). Particularly noteworthy are the subscales “affect tolerance” and “realistic object perception,” which distinguished best between AN-R and both AN-P and BN, as well as the subscales “experiencing affects” and “bodily self,” which were able to distinguish AN-P satisfactorily from AN-R and BN (Table 4).

### Strengths and Limitations

The present study systematically describes differences of personality structure facets across ED subtypes based on the OPD system for the first time. The OPD-SQ is based on a modern dimensional approach and although this instrument was not developed to assess DSM or ICD personality pathology, it has for example a large overlap with the DSM-5 levels of personality functioning (LPFS). However, due to its exploratory character and retrospective data analysis, this work is subject to a number of limitations: First, the small sample sizes of the subgroups resulted in a limited clinical significance and statistical power. The small sample size has probably led to borderline statistical significance in the large MANOVA model including 21 OPD-SQ subscales (2 of 4 test criteria did not reach statistical significance). Small effects might have missed statistical significance in all analyses. In addition, although samples sizes of subgroups are regarded as sufficient for discriminant analysis, larger samples have been recommended (Dunteman, 1984). Therefore, prospective studies using sufficiently large sample sizes are warranted in the future. Second, the mean BMI of the sample was lower as compared to other studies (Cuerda et al., 2019) which was the result from the specific clinical setting. As described above, lower weight affects brain structure and functions and these brain alterations may affect personality structure, too. Therefore, analyses in samples with a more representative mean weight are desirable in the future. Third, a bias could have been created by the assignment to the eating disorder subgroups on the basis of clinical ratings and medical reports. Again, this aspect should be considered in future prospective investigations with standardized diagnostic assessments using validated clinician-reported or patient-reported instruments. Moreover, the data were collected over a period of 5 years as part of clinical routine assessment which may have caused a bias. Last, the OPD-SQ instrument is a well-validated tool und broadly used in Germany. Although it has gathered international recognition over the last few years (Gazzillo et al., 2015; Kernberg, 2018), broad use is currently confined to German-speaking countries.

### Implications for Clinical Work and Research

As described above, differences observed in ED subtypes may be used to guide therapeutic interventions. Single scales may be used to discuss the aims of psychological treatments regarding personality functioning with patients. However, even using the OPD-SQ as an additional instrument, there are patients, which still cannot easily be assigned to a ED subgroup. This is visualized in Figure 3: There is a reasonable number of patients in the overlapping area in the center of the graph and it will still be challenging to assign these patients to a specific group. One reason for this problem may be the presupposition of ED subgroups as distinct disorders as classified in the diagnostic systems. Some researchers and clinicians believe, that eating disorder pathology should rather be conceptualized as a (uni-) dimensional construct and patients’ pathology may range between low ED pathology and severe ED pathology on a continuum (Wildes and Marcus, 2013). One of these approaches is supported, for example, by neurobiological correlates (Piccinni et al., 2015). Other researchers support a simple classification including four categories based on an analysis of data from the Eating Disorder Inventory (EDI) and BMI: AN-R, BN, BED and a category of patients who do not fully meet the criteria for any of the disorders (Sloan et al., 2005).

Most likely, there are many ways to classify eating disorders meaningfully, but attention should always be paid to their practicability and the additive benefits of classification. If the results of this study are confirmed, the OPD-SQ could be applied as a useful and practical additional tool for eating disorders. In this context, the application of the short version of the questionnaire (OPD-SQS), which has been developed and validated for screening (Ehrenthal et al., 2015; Obbarius et al., 2018), may also be appropriate. However, this needs to be investigated in future studies.

## Conclusion

Subgroups of EDs including AN-R, AN-P, and BN exhibit different patterns across their personality structure. Patients with an AN-R demonstrated a better level of personality structure as compared to patients with BN and AN-P, especially on the self-perception scale. Differences in personality structure can be successfully assessed by the self-report tool OPD-SQ. This instrument does not only seem to be suitable for the screening of personality functioning but can also support the diagnosis of different EDs and subtypes. However, due to its exploratory character, these findings need to be corroborated in larger prospective studies.

## Ethics Statement

This study was carried out in accordance with the recommendations of ICH-Guideline for Good Clinical Practice with written informed consent from all subjects. All subjects gave written informed consent in accordance with the Declaration of Helsinki. The protocol was approved by the institutional review board at Charité – Universitätsmedizin Berlin (EA4/011/18).

## Author Contributions

JR, AO, and MR contributed to the conception and design of the study. BV and JR were responsible for data assessment. JR organized the database, performed the statistical analysis, and wrote the first draft of the manuscript. JR, TH, and AO wrote sections of the manuscript. All authors contributed to manuscript revision, and read and approved the submitted version.

## Conflict of Interest

The authors declare that the research was conducted in the absence of any commercial or financial relationships that could be construed as a potential conflict of interest.

## References

[B1] Abbate-Daga G. Buzzichelli S. Amianto F. Rocca G. Marzola E. McClintock S. M. (2011). Cognitive flexibility in verbal and nonverbal domains and decision making in anorexia nervosa patients: a pilot study. *BMC Psychiatry* 11:162. 10.1186/1471-244X-11-162 21982555 PMC3199238

[B2] Al-Dajani N. Gralnick T. M. Bagby R. M. (2016). A psychometric review of the personality inventory for DSM-5 (PID-5): current status and future directions. *J. Pers. Assess.* 98 62–81. 10.1080/00223891.2015.1107572 26619968

[B3] American Psychiatric Association, (2013). *Diagnostic and Statistical Manual of Mental Disorders (DSM-5^®^).* Philadelphia: American Psychiatric Association.

[B4] Bender D. S. Zimmermann J. Huprich S. K. (2018). Introduction to the special series on the personality functioning component of the alternative DSM-5 model for personality disorders. *J. Pers. Assess.* 100 565–570. 10.1080/00223891.2018.1491856 30907715

[B5] Borenstein M. Hedges L. V. Higgins J. P. Rothstein H. R. (2011). *Introduction to Meta-Analysis.* Hoboken, NJ: John Wiley & Sons.

[B6] Brown T. A. Haedt-Matt A. A. Keel P. K. (2011). Personality pathology in purging disorder and bulimia nervosa. *Int. J. Eat. Disord.* 44 735–740. 10.1002/eat.20904 21321987

[B7] Cassin S. E. von Ranson K. M. (2005). Personality and eating disorders: a decade in review. *Clin. Psychol. Rev.* 25 895–916. 10.1016/j.cpr.2005.04.012 16099563

[B8] Cierpka M. Grande T. Rudolf G. von der Tann M. Staschf M. (2007). The operationalized psychodynamic diagnostics system: clinical relevance, reliability and validity. *Psychopathology* 40 209–220. 10.1159/000101363 17396047

[B9] Claes L. Vandereycken W. Luyten P. Soenens B. Pieters G. Vertommen H. (2006). Personality prototypes in eating disorders based on the big five model. *J. Pers. Disord.* 20 401–416. 10.1521/pedi.2006.20.4.401 16901262

[B10] Cuerda C. Vasiloglou M. F. Arhip L. (2019). Nutritional management and outcomes in malnourished medical inpatients: anorexia nervosa. *J. Clin. Med.* 8:E1042. 10.3390/jcm8071042 31319585 PMC6679071

[B11] Dinger U. Schauenburg H. Hörz S. Rentrop M. Komo-Lang M. Klinkerfuß M. (2014). Self- report and observer ratings of personality functioning: a study of the OPD system. *J. Pers. Assess.* 96 220–225. 10.1080/00223891.2013.828065 24003849

[B12] Dunteman G. H. (1984). *Introduction to Multivariate Analysis.* Thousand Oaks, CA: Sage Publications, Inc.

[B13] Ehrenthal J. C. Dinger U. Horsch L. Komo-Lang M. Klinkerfuß M. Grande T. (2012). Der OPD-Strukturfragebogen (OPD-SF): erste ergebnisse zu reliabilität und validität. *Psychother. Psychosom. Med. Psychol.* 62 25–32. 10.1055/s-0031-1295481 22271173

[B14] Ehrenthal J. C. Dinger U. Schauenburg H. Horsch L. Dahlbender R. W. Gierk B. (2015). Entwicklung einer zwölf-item-version des OPD-strukturfragebogens (OPD-SFK). *Z. Psychosom. Med. Psychother.* 61 262–274. 10.13109/zptm.2015.61.3.262 26388057

[B15] Ettl T. (2013). *Das Bulimische Syndrom: Psychodynamik und Genese.* Gießen: Psychosozial-Verlag.

[B16] Farstad S. M. McGeown L. M. Von Ranson K. M. (2016). Eating disorders and personality, 2004–2016: a systematic review and meta-analysis. *Clin. Psychol. Rev.* 46 91–105. 10.1016/j.cpr.2016.04.005

[B17] Fichter M. M. (1985). *Magersucht und Bulimia: Empirische Untersuchungen Zur Epidemiologie, Symptomatologie, Nosologie und Zum Verlauf.* Berlin: Springer. 3865053

[B18] Gabriel C. Waller G. (2014). Personality disorder cognitions in the eating disorders. *J. Nerv. Ment. Dis.* 202 172–176. 10.1097/NMD.0000000000000088 24469531

[B19] Gazzillo F. Lingiardi V. Del Corno F. Genova F. Bornstein R. F. Gordon R. M. (2015). Clinicians’ emotional responses and *Psychodynamic Diagnostic Manual* adult personality disorders: a clinically relevant empirical investigation. *Psychotherapy (Chic)* 52 238–246. 10.1037/a0038799 25868053

[B20] Gill J. (2000). *Generalized Linear Models: a Unified Approach.* Thousand Oaks, CA: Sage Publications.

[B21] Haslam N. Holland E. Kuppens P. (2012). Categories versus dimensions in personality and psychopathology: a quantitative review of taxometric research. *Psychol. Med.* 42 903–920. 10.1017/S0033291711001966 21939592

[B22] Jeammet P. (1997). Narzißtische und objektbezogene fehlregulierungen in der bulimie. *Psyche* 51 1–32.

[B23] Kask J. Ekselius L. Brandt L. Kollia N. Ekbom A. Papadopoulos F. C. (2016). Mortality in women with anorexia nervosa: the role of comorbid psychiatric disorders. *Psychosom. Med.* 78 910–919. 10.1097/PSY.0000000000000342 27136502

[B24] Kellett S. Hardy G. (2014). Treatment of paranoid personality disorder with cognitive analytic therapy: a mixed methods single case experimental design. *Clin. Psychol. Psychother.* 21 452–464. 10.1002/cpp.1845 23733739

[B25] Kernberg O. F. (2018). Commentary on the psychodynamic diagnostic manual, 2nd edition: what does the PDM-2 add to the current diagnostic panorama? *Psychoanal. Psychol.* 35 294–295. 10.1037/pap0000208

[B26] König K. Dahlbender R. W. Holzinger A. Topitz A. Doering S. (2016). Kreuzvalidierung von drei fragebögen zur strukturdiagnostik: BPI, IPO und OPD-SF. *Z. Psychosom. Med. Psychother.* 62 177–189. 10.13109/zptm.2016.62.2.177 27439554

[B27] Kroger C. Schweiger U. Sipos V. Kliem S. Arnold R. Schunert T. (2010). Dialectical behaviour therapy and an added cognitive behavioural treatment module for eating disorders in women with borderline personality disorder and anorexia nervosa or bulimia nervosa who failed to respond to previous treatments. An open trial with a 15-month follow-up. *J. Behav. Ther. Exp. Psychiatry* 41 381–388. 10.1016/j.jbtep.2010.04.001 20444442

[B28] Krug I. Root T. Bulik C. Granero R. Penelo E. Jimenez-Murcia S. (2011). Redefining phenotypes in eating disorders based on personality: a latent profile analysis. *Psychiatry Res.* 188 439–445. 10.1016/j.psychres.2011.05.026 21664698

[B29] Levy K. N. Clarkin J. F. Yeomans F. E. Scott L. N. Wasserman R. H. Kernberg O. F. (2006). The mechanisms of change in the treatment of borderline personality disorder with transference focused psychotherapy. *J. Clin. Psychol.* 62 481–501. 10.1002/jclp.20239 16470612

[B30] Martinussen M. Friborg O. Schmierer P. Kaiser S. Overgard K. T. Neunhoeffer A. L. (2017). The comorbidity of personality disorders in eating disorders: a meta-analysis. *Eat. Weight Disord.* 22 201–209. 10.1007/s40519-016-0345-x 27995489

[B31] McLachlan G. (2004). *Discriminant Analysis and Statistical Pattern Recognition.* Hoboken, NJ: John Wiley & Sons.

[B32] Miller G. A. Chapman J. P. (2001). Misunderstanding analysis of covariance. *J. Abnorm. Psychol.* 110 40–48. 10.1037//0021-843x.110.1.40 11261398

[B33] Murakami K. Tachi T. Washizuka T. Ikuta N. Miyake Y. (2002). A comparison of purging and non-purging eating disorder patients in comorbid personality disorders and psychopathology. *Tokai J. Exp. Clin. Med.* 27 9–19. 12472165

[B34] Obbarius A. Obbarius N. Fischer F. Liegl G. Rose M. (2018). Evaluation of factor structure and construct validity of the 12-item short version of the OPD structure questionnaire (OPD-SQS) in psychosomatic patients. *Psychother. Psychosom. Med. Psychol.* 69 38–48. 10.1055/s-0043-125394 29448281

[B35] Olson C. L. (1976). On choosing a test statistic in multivariate analysis of variance. *Psychol. Bull.* 83 579–586. 10.1037/0033-2909.83.4.579

[B36] OPD Task Force, (2008). *Operationalized Psychodynamic Diagnosis OPD-2: Manual of Diagnosis and Treatment Planning.* Göttingen: Hogrefe Publishing.

[B37] Paul T. Thiel A. (2005). *Eating Disorder Inventory-2 (EDI-2): Deutsche Version.* Göttingen: Hogrefe.

[B38] Peterhansel C. Linde K. Wagner B. Dietrich A. Kersting A. (2017). Subtypes of personality and ‘locus of control’ in bariatric patients and their effect on weight loss, eating disorder and depressive symptoms, and quality of life. *Eur. Eat. Disord. Rev.* 25 397–405. 10.1002/erv.2534 28719083

[B39] Pham-Scottez A. Huas C. Perez-Diaz F. Nordon C. Divac S. Dardennes R. (2012). Why do people with eating disorders drop out from inpatient treatment?: the role of personality factors. *J. Nerv. Ment. Dis.* 200 807–813. 10.1097/NMD.0b013e318266bbba 22922238

[B40] Piccinni A. Marazziti D. Vanelli F. Franceschini C. Baroni S. Costanzo D. (2015). Food addiction spectrum: a theoretical model from normality to eating and overeating disorders. *Curr. Med. Chem.* 22 1631–1638. 10.2174/0929867322666150227153015 25723508

[B41] Rosenvinge J. H. Martinussen M. Ostensen E. (2000). The comorbidity of eating disorders and personality disorders: a meta-analytic review of studies published between 1983 and 1998. *Eat. Weight Disord.* 5 52–61. 10.1007/bf03327480 10941603

[B42] Sansone R. A. Levitt J. L. Sansone L. A. (2004). The prevalence of personality disorders among those with eating disorders. *Eat. Disord.* 13 7–21. 10.1080/10640260590893593 16864328

[B43] Santonastaso P. Zanetti T. De Antoni C. Tenconi E. Favaro A. (2006). Anorexia nervosa patients with a prior history of bulimia nervosa. *Compr. Psychiatry* 47 519–522. 10.1016/j.comppsych.2006.02.003 17067877

[B44] Scharner S. Stengel A. (2019). Alterations of brain structure and functions in anorexia nervosa. *Clin. Nutr. Exp.* 44 1965–1975. 10.1016/j.yclnex.2019.02.001

[B45] Schmidt U. Treasure J. (2006). Anorexia nervosa: valued and visible. A cognitive-interpersonal maintenance model and its implications for research and practice. *Br. J. Clin. Psychol.* 45(Pt 3), 343–366. 10.1348/014466505x53902 17147101

[B46] Sheehan L. Nieweglowski K. Corrigan P. (2016). The stigma of personality disorders. *Curr. Psychiatry Rep.* 18:11. 10.1007/s11920-015-0654-1 26780206

[B47] Sloan D. M. Mizes J. S. Epstein E. M. (2005). Empirical classification of eating disorders. *Eat. Behav.* 6 53–62. 10.1016/j.eatbeh.2004.06.002 15567111

[B48] Stasch M. Cierpka M. Arbeitskreis Zur Operationalisierung Psychodynamischer Diagnostik, (2014). Operationalisierte psychodynamische Diagnostik OPD-2: das Manual für Diagnostik und Therapieplanung. Bern: Huber. 10.1016/j.eatbeh.2004.06.002

[B49] Thiel A. Jacobi C. Horstmann S. Paul T. Nutzinger D. O. Schüßler G. (1997). Eine deutschsprachige version des eating disorder inventory EDI-2 [German translation of the eating disorder inventory EDI-2]. *Psychother. Psychosom. Med. Psychol.* 47 365–376. 9411465

[B50] Thiel A. Paul T. (2006). Test–retest reliability of the eating disorder inventory 2. *J. Psychosom. Res.* 61 567–569. 10.1016/j.jpsychores.2006.02.015 17011367

[B51] Thompson-Brenner H. Eddy K. T. Franko D. L. Dorer D. J. Vashchenko M. Kass A. E. (2008). A personality classification system for eating disorders: a longitudinal study. *Compr. Psychiatry* 49 551–560. 10.1016/j.comppsych.2008.04.002 18970903

[B52] Treasure J. (2016). Eating disorders. *Medicine* 44 672–678.

[B53] Tyrer P. Crawford M. Mulder R. (2011). Reclassifying personality disorders. *Lancet* 377 1814–1815. 10.1016/S0140-6736(10)61926-5 21353696

[B54] Vall E. Wade T. D. (2015). Predictors of treatment outcome in individuals with eating disorders: a systematic review and meta-analysis. *Int. J. Eat. Disord.* 48 946–971. 10.1002/eat.22411 26171853

[B55] Waller G. Ormonde L. Kuteyi Y. (2013). Clusters of personality disorder cognitions in the eating disorders. *Eur. Eat. Disord. Rev.* 21 28–31. 10.1002/erv.2209 23080210

[B56] Westen D. Harnden-Fischer J. (2001). Personality profiles in eating disorders: rethinking the distinction between axis I and axis II. *Am. J. Psychiatry* 158 547–562. 10.1176/appi.ajp.158.4.547 11282688

[B57] Wildes J. E. Marcus M. D. (2013). Incorporating dimensions into the classification of eating disorders: three models and their implications for research and clinical practice. *Int. J. Eat. Disord.* 46 396–403. 10.1002/eat.22091 23658078 PMC3744318

[B58] World Health Organization [WHO] (1993). *The ICD-10 Classification Of mental and Behavioural Disorders: Diagnostic Criteria for Research.* Geneva: World Health Organization.

[B59] World Health Organization [WHO] (2018). *International Classification of Diseases for Mortality and Morbidity Statistics (11th Revision).* Geneva: World Health Organization.

[B60] Wright A. G. Simms L. J. (2014). On the structure of personality disorder traits: conjoint analyses of the CAT-PD, PID-5, and NEO-PI-3 trait models. *Pers. Disord.* 5 43–54. 10.1037/per0000037 24588061 PMC3942782

[B61] Zimmermann J. Dahlbender R. Herbold W. Krasnow K. Turrión C. Zika M. (2015). Der OPD- Strukturfragebogen erfasst die allgemeinen merkmale einer persönlichkeitsstörung. *Psychother. Psychosom. Med. Psychol.* 65 81–83. 10.1055/s-0034-1395626 25503590

[B62] Zimmermann J. Ehrenthal J. C. Cierpka M. Schauenburg H. Doering S. Benecke C. (2012). Assessing the level of structural integration using operationalized psychodynamic diagnosis (OPD): implications for DSM–5. *J. Pers. Assess.* 94 522–532. 10.1080/00223891.2012.700664 22808938

